# The Medico-Chirurgical Review and Journal of Practical Medicine

**Published:** 1847-08

**Authors:** 


### ARTICLE XVI.

*The Medico-Chirurgical Review and Journal of Practical Medicine.* January, 1847. New York: Republished by R. and G. S. Wood. (In Exchange.)

This work, which has for so many years held a rank among the highest critical authorities, still retains its high character and interest, and furnishes in itself so perfect an analysis of current medical literature, as to render unnecessary, in many instances, a reference to the works reviewed. The republication, as will be seen, is continued by the Messrs. Woods, in New York. D. B.

